# Energy Structure and Energy Security under Climate Mitigation Scenarios in China

**DOI:** 10.1371/journal.pone.0144884

**Published:** 2015-12-10

**Authors:** Ken’ichi Matsumoto

**Affiliations:** School of Environmental Science, The University of Shiga Prefecture, Hikone, Shiga, Japan; Universidade de Vigo, SPAIN

## Abstract

This study investigates how energy structure and energy security in China will change in the future under climate mitigation policy scenarios using Representative Concentration Pathways in a computable general equilibrium model. The findings suggest that to reduce greenhouse gas emissions, China needs to shift its energy structure from fossil fuel dominance to renewables and nuclear. The lower the allowable emissions, the larger the shifts required. Among fossil fuels, coal use particularly must significantly decrease. Such structural shifts will improve energy self-sufficiency, thus enhancing energy security. Under the policy scenarios, energy-source diversity as measured by the Herfindahl Index improves until 2050, after which diversity declines because of high dependence on a specific energy source (nuclear and biomass). Overall, however, it is revealed that energy security improves along with progress in climate mitigation. These improvements will also contribute to the economy by reducing energy procurement risks.

## Introduction

Climate change is currently the most significant global environmental issue, and climate-related policy discussions with mid- to long-term perspectives are ongoing worldwide. A critical negotiated treaty is the United Nations Framework Convention on Climate Change (UNFCCC). The Copenhagen Accord of December 2009 was an important step along the global path to climate change. The Annex I parties and some major non-Annex I parties of the UNFCCC, such as China and India, submitted their pledges on greenhouse gas (GHG) emission reduction by the end of January 2010. The Kyoto Protocol expired at the end of 2012, but at the 2012 Conference of the Parties to the UNFCCC, there was agreement to extend the Protocol until 2020 and to develop a successor by 2015. However, important developed countries such as Japan and Russia did not participate in the second commitment period of the Kyoto Protocol.

Energy demand has dramatically increased in recent years in large emerging countries such as China and India. This demand, driven by economic and population growth, is expected to increase further [1–2], raising concerns about energy supplies in the future. In addition, because production and reserves of fossil fuels such as crude oil and natural gas are predominately located in a small number of countries [1], countries that are poor in energy resources and dependent on imported fossil fuels will face potential price fluctuations and geopolitical risks.

Climate change measures, of which only mitigation measures are considered in this study, are aimed at reducing GHG emissions, in particular carbon dioxide (CO_2_). To emit less GHGs, promotion of energy efficiency and shifts to low-carbon energy, namely shifts from coal to natural gas and from fossil fuels to renewables and nuclear energy, are critical. That is, energy structure is expected to change drastically. In addition, if energy savings and low-carbon energy use are adopted as climate change measures, the volume of and dependence on imported energy will decrease. Renewables are basically domestic energy and nuclear energy is considered a semi-domestic energy [3–4]. This in turn will help improve energy security [5–6].

To achieve energy security in countries that rely on foreign energy sources, risk diversification is essential. Methods include diversifying supply (importing fuels from a larger number of countries), diversifying fuel types, and diversifying locations of production (industrial globalization) [7]. It is also important to reduce energy imports and to increase energy self-sufficiency for energy security. It should be noted that reducing energy imports does not necessarily link to welfare improvement because of the comparative advantage theory. In case energy is imported, importing from secure countries lowers the risk.

In this context, China has a significant energy security issue. China produces fossil fuels, but its demand exceeds its production [1]. In addition, it is expected that China will continue to experience high economic growth in the future, meaning that energy demand and energy imports will both increase significantly [2].

Research on energy security in China is a component of the Asian Energy Security Project [8], coordinated by the Nautilus Institute [9]. The project uses both a narrow definition of energy security, referring only to energy supply, and a broader definition, referring to energy supply and accompanying economic, technological, environmental, social, cultural, and military perspectives. The project utilizes the Long-range Energy Alternatives Planning (LEAP) software system [10–12], a scenario and energy pathway modeling tool that enables analysis of possible energy systems, to analyze energy security in Asia. Energy pathways or scenarios are internally consistent storylines of how an energy system might evolve over time (in the short to medium term) in a particular socioeconomic setting and under a particular set of policy conditions [11–12]. Multiple energy pathways within a country are compared to indicate which pathway is preferable according to various energy security criteria such as cost, energy output, fuel imports and exports, and technological development. Other external methods, such as diversification indices, multiple-attribute analysis and matrices, and qualitative analysis, can be applied using the results from the LEAP system for further analysis of energy security [11]. For example, one study in the project analyzed three scenarios focusing on the deployment of nuclear power until 2030 from the perspective of the structure of power generation and GHG emissions in China [8]. Because the LEAP system is a bottom-up energy model, detailed modeling of energy technology is conducted. However, it does not consider industrial sectors other than energy sectors nor interactions among industrial sectors. It is also important to consider climate change and energy issues simultaneously on a global basis when making inferences about future energy situations.

In this paper, the author focuses on the energy structure and energy security issues in China. Energy security is an important issue for the country because its economic activities are highly dependent on fossil fuels and it is one of the world’s largest fossil fuel importing countries [1–2]. In addition, China is highly dependent on the Middle East for oil.

In this study, energy structure and energy security under climate mitigation policies are examined using a computable general equilibrium (CGE) model. By using a CGE model, an inclusive assessment of economic activities (markets), including energy sectors and non-energy sectors, and all economic activities of a country are kept in the analysis, an approach that differs from the abovementioned studies. In addition, CGE allows multiple climate mitigation scenarios to be considered simultaneously for comparison. CGE is a well-known model for evaluating climate and energy policies, and it is also frequently applied to China [13–20]. The model application to China includes evaluation of the economic impact of its emission reduction or low-carbon pathways (including both domestic targets and international pledge) [13–15,18–19], the economic impact of carbon tariffs [17], international technology diffusion under climate mitigation policy [16], and the economic impact of an emissions trading system [20]. However, this kind of model has not been applied to the examination of energy security issues.

In the literature, there is no consensus on the definition of energy security [21]. For example, some studies focus on the aspect of energy supply, but others use more comprehensive concepts, including social, economic, and/or environmental impacts [10–11,21]. According to comprehensive reviews of energy security [21], one or more of seven energy security dimensions (i.e., availability, infrastructure, prices, social effects, environment, governance, and efficiency) have frequently been used in the literature. Furthermore, the number of indicators used in the literature varies from one to a few hundred. Concerning the weights for aggregating multiple dimensions (if aggregation is performed), equal weights are used in some studies, while different weights, such as fuel share, are used in the others [21].

This study uses one of the simplest definitions of energy security, i.e., energy supply, which is the aspect most frequently used in the literature [21]. The key to energy security is a stable supply of energy. One of the ways to achieve a stable supply is diversification (balance) of energy types [7], since if an energy source is lost for some reason, the probability of avoiding or reducing the loss will increase. Even for renewables, which are domestic energy sources, more is not always better for energy security, since their output is affected by meteorological (weather) and climatic factors (e.g., solar radiation for PV and precipitation for hydropower, and climate change which changes climate conditions in the future) [22–23]. Weather variability is key to the cause of solar and wind energy generation variability [24], and the potentials of renewable energy sources will be impacted by climate change in many regions [25]. Basically, meteorological factors are short-term influences, while climatic factors are long-term ones [26,27]. However, considering energy security, which is a long-term issue [28], both factors affect use of renewables, because climate change affects meteorological conditions. The second point is diversifying energy import, including tradeoffs between importing and domestic supply. To reduce the risk and improve energy security, a lower amount of energy imports is preferable. Furthermore, diversifying the origins of energy imports is also preferable when importing energy.

In this study, changes in primary and final energy demand are first investigated. Energy security in terms of primary energy structure, diversity of energy type, and net imports of fossil fuels (origin of energy imports are not considered in this study. See below for the reason) are then analyzed. In addition, the analysis is extended to the entire 21^st^ century, in contrast to previous research, which focused only on the short-to-medium term. The long-term consequences are important, because the world will be highly dependent on fossil fuels for many years to come [29–32]. There exist studies that aggregate multiple indicators into one to explore energy security. However, this study does use the two indicators (i.e., the diversity of energy type and imported energy) separately (but comprehensively), since there is also no consensus on the way for weighting the multiple indicators and many studies that do not aggregate them exist [21].

The other important aspects in analyzing a stable supply of energy are the origin of energy import and country risks of energy exporters [33–34] as mentioned above. However, these aspects are not applicable in this study since import by origin is not available from the model results and future country risks are unpredictable. Therefore, the structure and balance of energy types, as well as the import of energy (total in the country rather than by origin), are separately shown in this study, instead of aggregating them as in the two previously-mentioned studies [33–34].

## Methods

### Model

A CGE model was used to evaluate how China’s energy security and energy structure change under climate mitigation scenarios during the 21^st^ century. This model is based on the work done by the author and his colleagues [29,35–38]. In contrast to bottom-up LEAP modeling, a CGE model is a top-down model often used for analyzing the economic implications of energy and climate change issues and policy designs [13–20,36–37,39–41].

**Fig 1:**
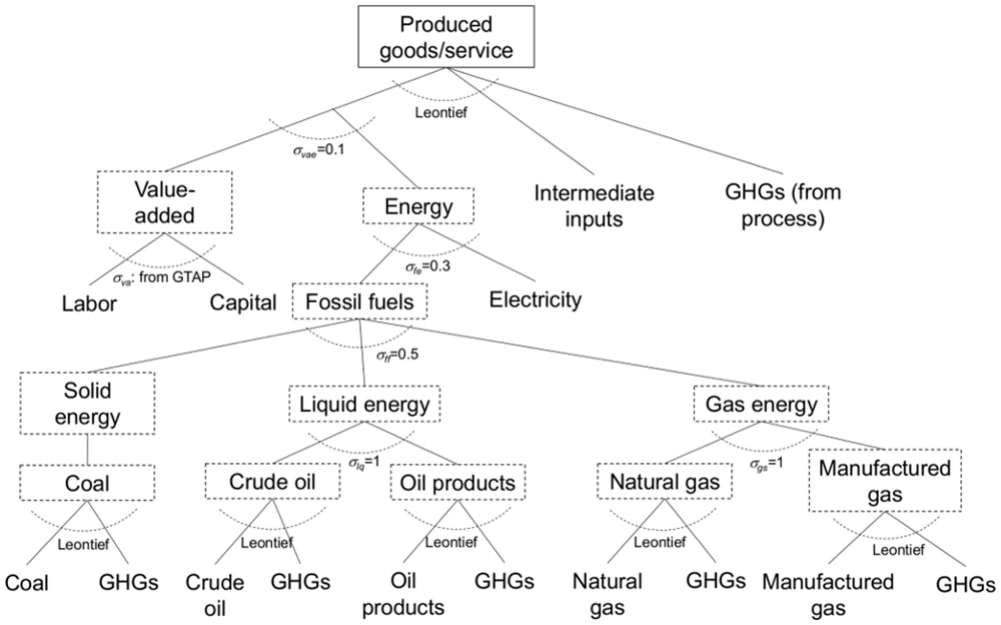
Production structure for the manufacture and service sectors (EIS, M_M, OMF, FOD, CNS, TRT, CMN, WTR, OSG, and SER). This figure shows the most basic structure in the model. In each nest, corresponding factors are aggregated by a CES function.

This study applied a multi-regional and multi-sectoral recursive dynamic CGE model on a global scale, combining energy and environmental components. Model details are explained in the author’s previous study [42], but an overview is provided here. The model consists of 24 geographical regions (Table 1), each producing 21 economic goods and services (Table 2) and each having a final demand sector. One sector produces one type of goods or services, and it is assumed that all markets are perfectly competitive and that production is subject to “constant returns to scale” technology. In the energy sectors, electric power is disaggregated into specific production technologies, including thermal, hydro-, and nuclear power and renewables (see Table 2 for the details). Carbon capture and storage (CCS) technology can be designated an advanced technology for thermal and biomass power generation. Each industrial sector is represented by a nested constant elasticity of substitution (CES) production function (e.g., Fig 1). Although all of the production structures are based on nested CES functions, several different production structures are assumed for different sectors. Fig 1 shows the most basic structure in which each type of goods or service is produced as a Leontief aggregate of a value-added and energy composite and intermediate inputs, and is applied to several of the defined sectors. The value-added and energy composite is a CES aggregate of the value added and the energy input composite. The value added is a CES aggregate of labor and capital. The energy composite is a CES aggregate of a fossil fuels composite and electricity. The fossil fuels composite is a CES aggregate of coal, a liquid energy composite, and a gas energy composite. The liquid energy composite and the gas energy composite are CES aggregates of crude oil and petroleum products, and of natural gas and gas manufacture and distribution, respectively. During production, GHGs are emitted from fossil fuels and industrial processes. Fossil fuel emissions are considered as Leontief aggregates at the bottom-level nests, while industrial emissions are considered as the Leontief aggregate at the top-level nest.

**Table 1:** Region definitions.

Code	Region
AUS	Australia
NZL	New Zealand
JPN	Japan
CAN	Canada
USA	United States of America
E15	15 Western EU countries
RUS	Russia
E10	10 Eastern EU countries
XRE	Other Europe (e.g., Bulgaria)
KOR	South Korea
CHN	China and Hong Kong
XRA	Other Asia-Pacific (e.g., Mongolia)
IDN	Indonesia
THA	Thailand
XSE	Other Southeast Asia (e.g., Malaysia)
IND	India
XSA	Other South Asia (e.g., Bangladesh)
MEX	Mexico
ARG	Argentina
BRA	Brazil
XLM	Other Latin America (e.g., Chile)
XME	The Middle East (e.g., Saudi Arabia)
ZAF	South Africa
XAF	Other Africa (e.g., Egypt)

**Table 2:** Commodities/sectors definitions.

Code	Commodities/sectors
Energy commodities/sectors	
COA	Coal
OIL	Crude oil
GAS	Natural gas
P_C	Petroleum products
GDT	Gas manufacture and distribution
ELY	Electric power ^a^
Non-energy commodities/sectors	
AGR	Agriculture (e.g., rice)
LVK	Livestock (e.g., cattle)
FRS	Forestry
FSH	Fishery
EIS	Energy-intensive industries (e.g., chemical products)
OMN	Other mineral mining
M_M	Metals and manufacturing (e.g., motor vehicles)
FOD	Food processing (e.g., food products)
OMF	Other manufacturing (e.g., textiles)
CNS	Construction
TRT	Transportation (e.g., air transportation)
CMN	Communication
WTR	Water
OSG	Governmental services (e.g., education)
SER	Other services (e.g., insurance)

^a^ The electric power sector consists of thermal power (i.e., coal-, oil-, and gas-fired), nuclear power, hydropower, solar power, wind power, geothermal power, biomass power, waste power, and other renewables. In addition, thermal power and biomass power with CCS technology are included in the analysis.

In examining the fishery and other mineral-mining sectors, resources (natural resources) are treated as a component of the value added. Similarly, in examining the agriculture, livestock, and forestry sectors, land is treated as a component of the value added.

In the production structure for the fossil fuels extraction sectors, resources are considered to be aggregated at the top-level nest. In the model, the magnitude of the resource limits and associated extraction costs are taken into account [43].

With respect to the petroleum products sector, crude oil is considered to be aggregated at the top-level nest (and not treated as energy), because most crude oil is used as feedstock in this sector. Similarly, in the gas manufacture and distribution sector, natural gas is considered to be aggregated at the top-level nest to treat it as feedstock in this sector.

Finally, there is a slightly different structure in the electricity sector. The thermal power sectors use corresponding fossil fuels as an input (e.g., coal for coal power generation), while the renewables sectors do not. However, the biomass power sector uses land as an input, and the other renewables sectors use the input of their corresponding renewable sources. This structure is similar to the Emissions Prediction and Policy Analysis model [44].

Each industrial sector produces goods and services for international and/or domestic markets. The Armington assumption [45] is applied for international trade. In the model, goods/services from different regions are aggregated through a two-stage CES function; first, imports from different regions are aggregated into a composite import and then a composite import and domestic goods/services are aggregated.

In each domestic market, the supplied goods and services are consumed as final consumption, investment, and/or intermediate input for industrial sectors. The total investment demand in each period, which forms capital in the next period, is set exogenously to meet a prescribed future economic growth rate (see Section 2.2.1). In more precise terms, economic growth is realized by increasing production factors (capital, labor, land, and resources) and improving efficiency (including energy efficiency, land productivity, and total factor productivity). In the model, investment demand is assumed to increase in line with the increased rate of Gross Domestic Product (GDP). Since the model used here is of a recursive dynamic type, the total investment demand cannot be determined endogenously. In addition, the model uses a putty-clay approach for forming capital. It includes two types of capital, existing capital and new capital. Existing capital cannot be moved among sectors, while new capital can be installed in any sector. However, new capital is subsequently handled as existing capital when it is installed in a certain sector. Technological improvements are applied only to new capital. Thus, the productivity of aggregated (existing and new) capital is the weighted average of the technology levels in existing and new capital. This suggests that the more new capital is installed, the more rapid the efficiency change will be.

The final demand sector in each region owns all production factors (i.e., capital, labor, land, and resources) and supplies them to the industrial sectors to generate income of the final demand sector for final consumption and savings. The final demand for each good or service is determined in order to maximize the utility represented by the relevant CES function in each period. GHGs are emitted when the final demand sector consumes fossil fuels.

The model endogenously handles the global emissions of 10 gases, including CO_2_, and is run to follow the emission pathways described in Section 2.2.2 between 2001, the base year, and 2100.

Global-scale GHG emissions trading is considered in the model, which assumes that GHG emissions are reduced cost efficiently. The global GHG emissions are assigned to regions in proportion to the regions’ projected populations from the year 2050 onwards. Between the base year and 2050, regional GHG emission limits were set by linear interpolation of emissions in these years (i.e., Contraction and Convergence).

The model was calibrated to reproduce economic activity, energy, and GHG levels in the base year using the following data: the Global Trade Analysis Project (GTAP) version 6 database [46] for economic activity levels; the Emission Database for Global Atmospheric Research (EDGAR) version 4 database [47] for GHG emissions; and the IEA energy balance tables [48–49] for energy. The GTAP database used here is not the latest version. However, the older version is selected in this study because it was developed based on data from 2001, which almost corresponds to the base year of the Representative Concentration Pathways (RCPs), the policy scenarios used in this study (see Section 2.2.2).

### Scenarios

Energy security was analyzed by applying the reference scenario and policy scenarios to the CGE model.

#### Reference scenario

As a first step in the process of developing policy scenarios (see Section 2.2.2), a business-as-usual scenario, or a reference scenario, was developed. Under this “no-climate-policy” scenario, GHG emissions and concentrations, and radiative forcing would be expected to exceed those of the policy scenarios. The reference scenario assumes that no policies and measures solely aimed at controlling GHG emissions are introduced, beyond those already in place, and that the existing policies are not renewed when they expire. The reference scenario makes several assumptions. Demographic assumptions are based on a medium variant of the UN World Population Prospects [50–51]. Future economic growth assumptions are based on the Sustainability First scenario presented [52]. Finally, technological improvement assumptions are based on the Special Report on Emission Scenarios (SRES) B2 scenario [53]. The SRES B2 scenario was selected because it is a moderate scenario, and the population and GDP are similar to the assumptions in this study [53].

**Fig 2:**
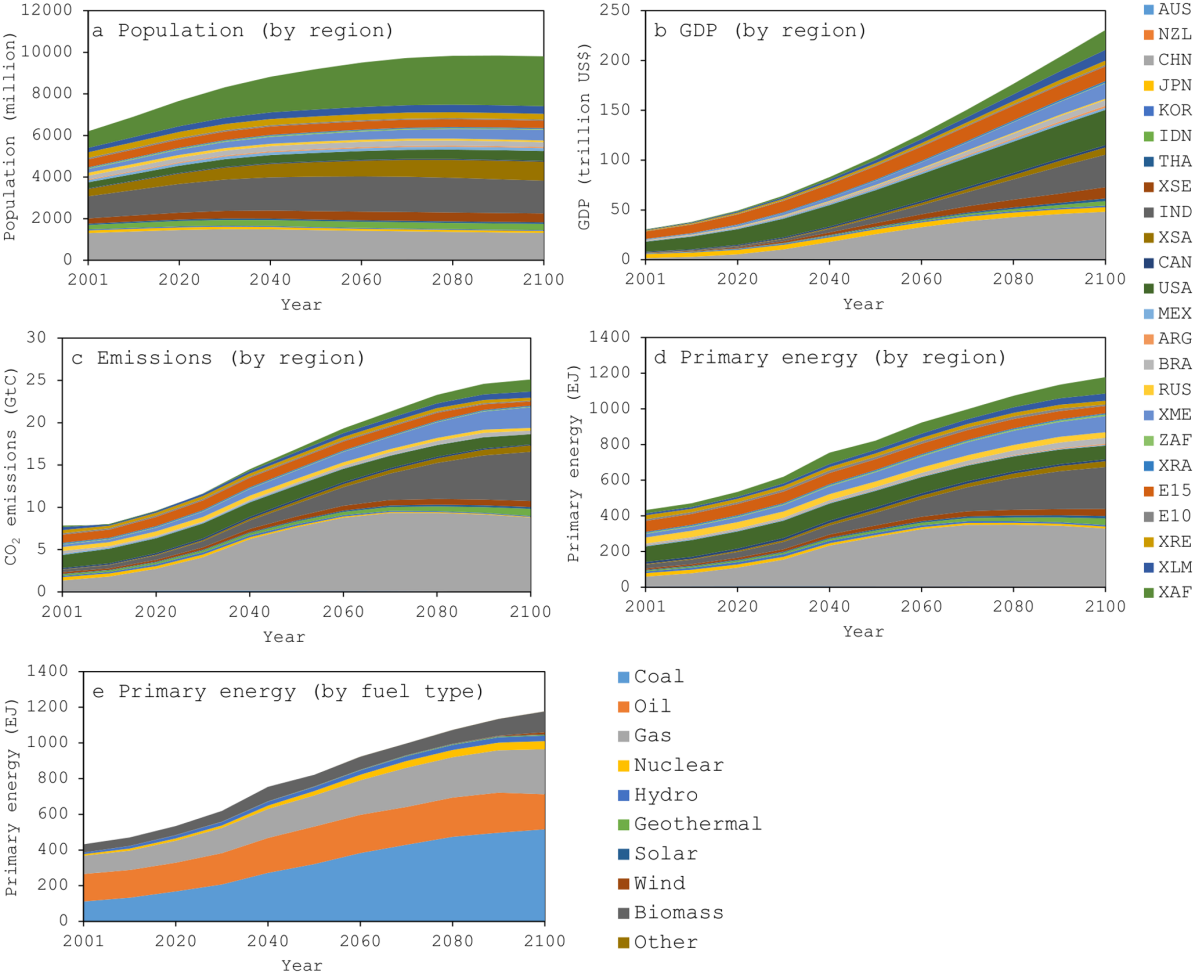
Characteristics of the reference scenario. (a) Population. (b) GDP. (c) Total CO_2_ emissions. (d) Primary energy demand by region. (e) Primary energy demand by fuel type.

Fig 2 summarizes the reference scenario. As the figure indicates, the model reveals that global population, global economy in terms of GDP, global energy demand (particularly coal), and global CO_2_ emissions will increase in the 21^st^ century.

#### Policy scenarios

RCPs are used for the climate change policy (mitigation) scenarios [54–55]. RCPs are the first step toward the Fifth Assessment Report of the Intergovernmental Panel on Climate Change (AR5) [56] and one of the latest climate policy scenario families. RCPs are defined by radiative forcing levels in 2100 and consist of four scenarios: the lowest 2.6 W/m^2^ [32], the highest 8.5 W/m^2^ [30], and the two middle scenarios of 4.5 W/m^2^ [31] and 6 W/m^2^ [29].

**Fig 3:**
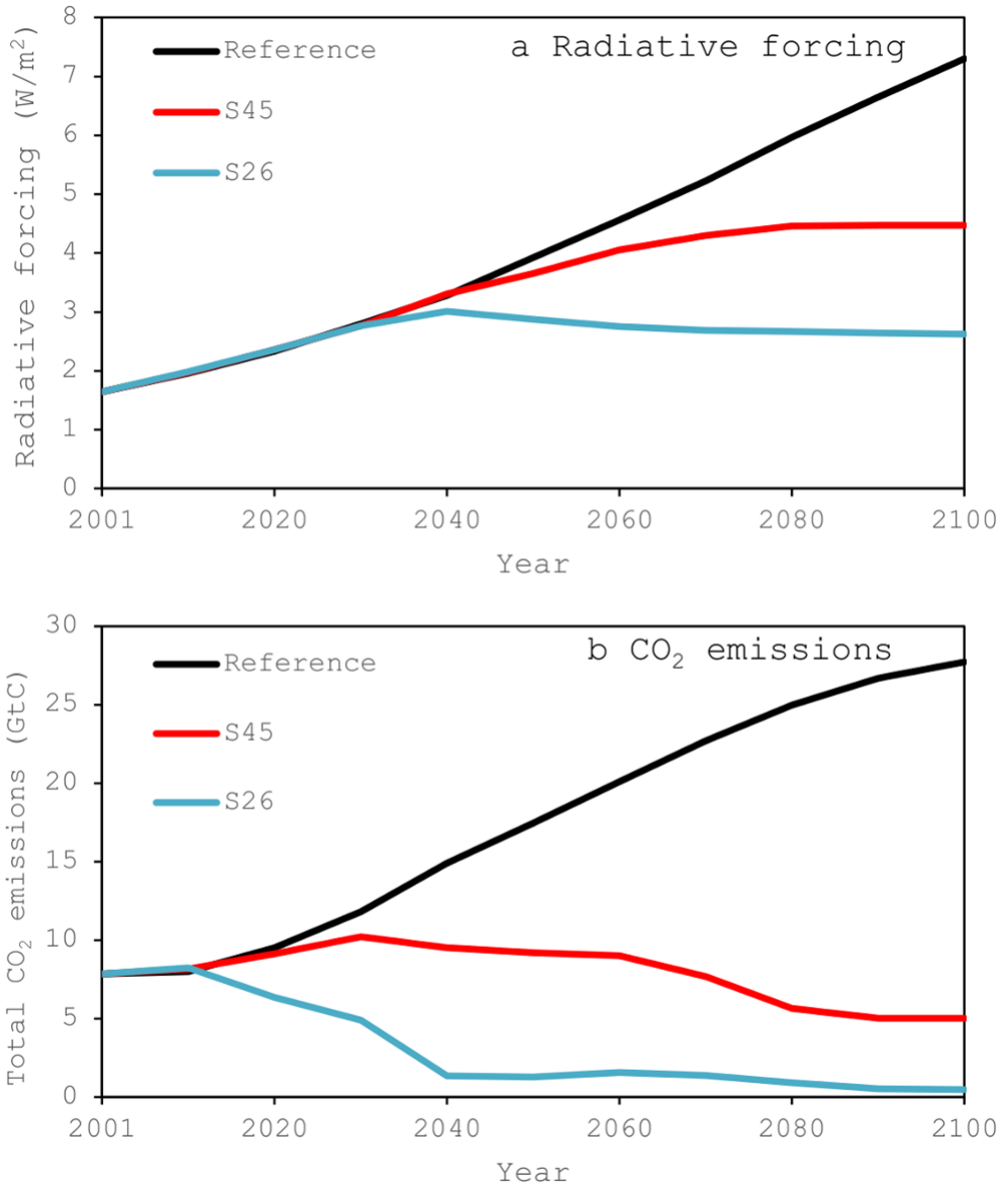
Radiative forcing levels and corresponding total CO_2_ emissions in the scenarios. (a) Radiative forcing. (b) CO_2_ emissions. Both figures are created based on the author’s previous study [35].

Using the CGE model, the author analyzed the medium-low scenario (S45 below), with RCP 4.5 W/m^2^, and the lowest scenario (S26 below), with RCP 2.6 W/m^2^, and compared them to the reference scenario (Fig 3). Since this study examines the energy security issue under climate mitigation scenarios, it is preferable to use relatively new scenarios in this area. Thus, the RCP scenarios used in the AR5 [56] and other recent studies in this area [57–58] were chosen. Furthermore, since the emissions of the highest RCP 8.5 W/m^2^ scenario [30] exceed those of the reference scenario, and the climate mitigation level of the RCP 6 W/m^2^ is much higher than that required for achieving the ultimate objective of the UNFCCC [29,55], the S45 and S26 scenarios were selected for this study.

Note that the energy structure is not exogenously controlled to maintain a specific level or percentage (such as *p*% or *e* GWh of nuclear power generation in country *A* in year *Y*) when simulating either the reference or policy scenarios. The model analyzes endogenously changing energy structure and energy security according to the scenarios.

## Results and Discussion

To understand changes in energy structure and energy security under climate change policies in China, the author focused on changes in primary energy demand and final energy demand, fossil fuel imports, and diversity of energy types in the 21^st^ century. In this section, the results in years 2050 and 2100 are mainly discussed. It should be noted that this study focuses on the relationship between climate mitigation and energy structure and security, while the relationship between energy structure/security and welfare is out of the scope.

**Fig 4:**
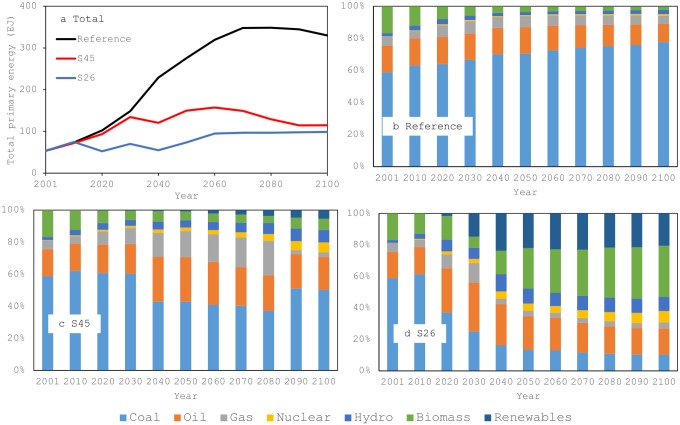
Total primary energy demand and the energy structure. (a) Total primary energy demand. (b) Energy structure for the reference scenario. (c) Energy structure for the S45 scenario. (d) Energy structure for S26 scenario. Renewables in Panels (b)–(d) include those listed in the footnote to Table 2, except hydropower and biomass.

In the reference scenario, the total primary energy demand increases in this century (Fig 4a). It is 53.5 EJ in the base year but increases to 275.6 EJ in 2050 and 330.0 EJ in 2100. Economic development and a growing population drive the increasing primary energy demand. In the two policy scenarios, the total primary energy demand is smaller than in the reference scenario, with the demand in the S26 scenario the smallest. This means that reducing the total primary energy demand is required to reduce GHG emissions from the reference scenario levels in this study. It should be noted that even under the S26 scenario, the primary energy demand exceeds the base year level.

The energy structure drastically changes in China over the study period (Fig 4b–4d). It is important to note that energy structure is not controlled exogenously in the model, but is determined by the relative cost of each energy source in the model as previously described. Under the reference scenario, the percentage of fossil fuels in the total primary energy demand increases and exceeds 90% in the middle of this century, with coal—the cheapest energy—the main fuel source.

The policy scenarios have demand for fossil fuels, especially coal, significantly decreasing in the country, and use of renewables and nuclear energy increasing. In China, the percentage of renewables, including biomass power, increases significantly, especially in the S26 scenario and in the later years of the study period in both scenarios. In the model, the available land for agriculture is based on the GTAP database [46], taking into account possible competition in land use among sectors that use land as a production factor, including biomass energy (bio-energy crops) [29,46]. For future land use, land productivity improvement is also assumed [29]. In addition, for future renewables, their potential is assumed based on the survey implemented by the National Institute for Environmental Studies [59]. In these assumptions, the land mass of a region is an important factor for determining potential, since the larger the land area, the larger the areas for cultivating bio-energy crops and installing renewable-energy facilities. China’s potential to harness renewables from its enormous land mass is promising. The absolute amount of nuclear energy in the policy scenarios is increasing, but the percentage is not increasing largely because of China’s high primary energy demand.

**Fig 5:**
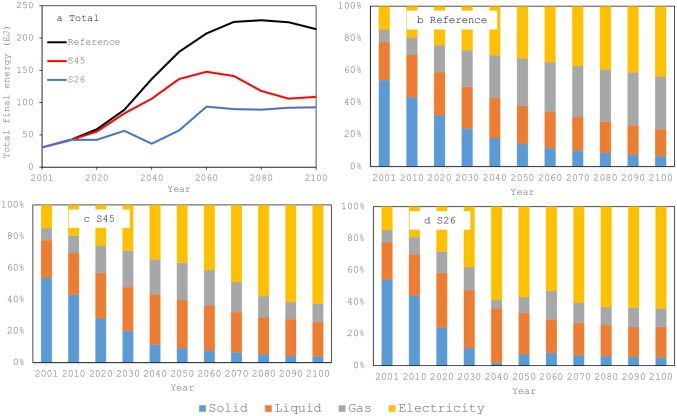
Total final energy demand and the energy structure. (a) Total final energy demand. (b) Energy structure for the reference scenario. (c) Energy structure for the S45 scenario. (d) Energy structure for S26 scenario.

The trends observed in the total final energy are similar to the total primary energy (Fig 5a). In the reference scenario, the total final energy demand increases in this century. It is 30.6 EJ in the base year but increases to 178.6 EJ in 2050 and 213.9 EJ in 2100. In the two policy scenarios, the total final energy demand is smaller than in the reference scenario, with the demand in the S26 scenario the smallest. However, it should be noted again that even under the S26 scenario, the final energy demand exceeds the base year level. The energy structure drastically changes over the study period (Fig 5b–5d), with similar trends in the three scenarios. In each scenario, electrification of China progresses in the future. Because coal use in primary energy increases in the reference scenario (Fig 3), power generation from coal-fired power plants also increases. The percentage of electricity is higher in the policy scenarios than in the reference scenario. In these cases, more renewables and biomass power are used for power generation.

**Fig 6:**
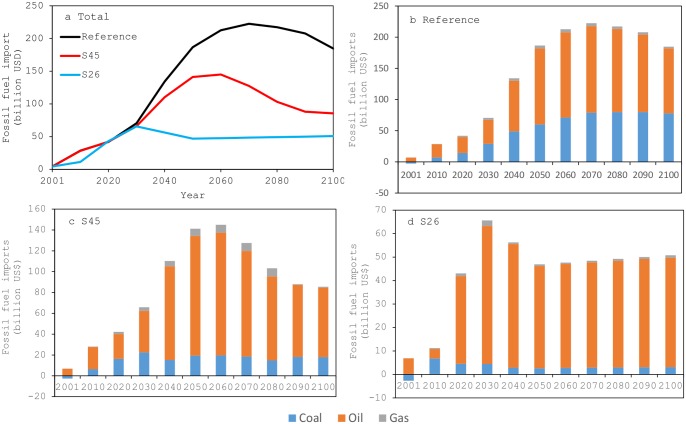
Net import of fossil fuels. (a) Total net import. (b) Breakdowns of the net import for the reference scenario. (c) Breakdowns of the net import for the S45 scenario. (d) Breakdowns of the net import for the S26 scenario.

With respect to trade activity of fossil fuels in terms of net imports (imports minus exports) (Fig 6), the total steadily increases in the 21^st^ century. Although China is a net-exporting country of coal in the base year, it soon becomes a net-importing country in both the reference and policy scenarios.

Under the S45 scenario, the net imports of fossil fuels relative to the reference scenario are 24.4% lower by 2050. A greater decline of 74.9% is seen under the S26 scenario. By 2100, the net fossil fuel imports under the S45 scenario have declined from the reference case by 53.7%. Under the S26 scenario, the reduction is 72.5%, which is lower than the S45 scenario but slightly higher than the 2050 level. This is due to the introduction of more coal power with CCS technology in the latter half of the century.

Considering the net imports by fuel type, the decline in coal is the largest, followed by oil and natural gas. The lower-emission scenarios tend to show lower net imports for all types, except for natural gas in around 2050 under the S45 scenario. Because the carbon intensity of natural gas is the lowest of the fossil fuels, the use of natural gas is often promoted to reduce GHG emissions. However, to reduce GHG emissions to targets for the latter half of the 21^st^ century in the S45 scenario and also for the S26 scenario, shifting to low-carbon-intensive fossil fuels is not sufficient, and reducing the total amount of fossil fuel use will be required.

The study indicates that decreasing dependence on fossil fuels and increasing the amount and percentage of renewables and nuclear energy will help to mitigate climate change. China is a net importer of fossil fuels, and its dependence is very high. Replacing fossil fuels and reducing China’s net imports will improve energy self-sufficiency and improve energy security, an important consequence of promoting climate change measures.

As Fig 4 illustrates, the dependence on fossil fuels, which are largely imported energy sources, decreases significantly in the policy scenarios, especially under the S26 scenario. Similarly, net imports of fossil fuels decrease under the policy scenarios (Fig 6), underlining the important roles of reducing imported energy and increasing the rate of self-sufficiency in achieving energy security. Increasing the diversity of energy sources (primary energy) is also important, as this can diversify the risk [7]. The Herfindahl Index (HI) is a metric for the energy security implications of different patterns of energy supply and demand. It is based on diversity indices used in the economic and financial analysis [7,10,60] and is applied to measure the effects of diversification of energy sources. The index has a maximum value of one when there is only one energy source and decreases with additional energy sources. The lower the value of the index, the more diverse the sources. When calculating the index (eq 1), renewables are disaggregated by type—namely solar, wind, geothermal, and other renewables (see Table 2 and Fig 4).

$$H=\sum_{i}x_{i}^{2}$$ (1)

where *H*: Herfindahl Index, and *x*_*i*_: the fraction of primary energy demand by energy type *i*.

**Fig 7:**
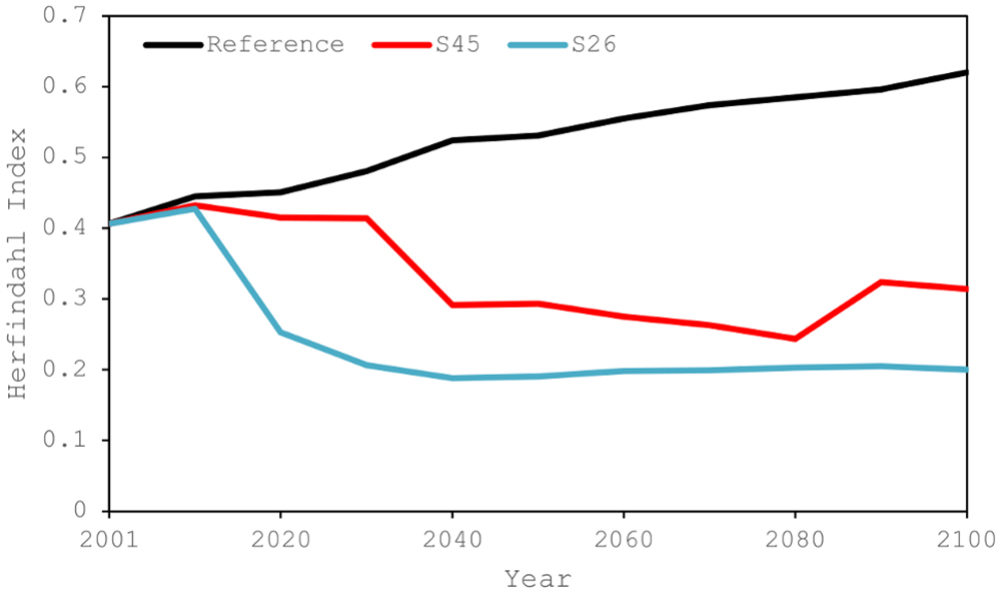
Herfindahl Indices showing trends in energy-source diversity under a reference and two climate-mitigation scenarios.

Applying the index to the scenarios, the values change over time relative to the base year (Fig 7). In the reference scenario, diversity worsens over time because China depends more heavily on coal in the future.

In comparison to the reference scenario, there is an increase in the diversity of energy sources by the middle of this century under the policy scenarios. However, the diversity declines toward the end of this century in both the S45 and S26 scenarios, although it is better than the base year. The HI value is smaller in the S26 scenario, indicating that when reducing GHG emissions as part of climate change mitigation, the diversity of energy sources initially improves by reducing dependence on fossil fuels, especially coal. Diversity then tends to retract as China concentrates on biomass and nuclear energy. Although the HI values become higher in 2100 than in the middle of this century (Fig 7), renewable energy technologies and other future potential technological breakthroughs can improve the diversity of energy sources.

The results suggest that energy structure, for both primary energy and final energy, changes markedly under climate mitigation scenarios. In addition, energy security is improved under the scenarios by reducing fossil fuel imports and increasing energy self-sufficiency, a consequence of a reduction in total primary energy demand and a shift in energy structure from fossil fuels. However, the impact on diversity of energy sources differs by scenario, depending on which types of energy sources are selected when shifting from fossil fuels, how fast such a shift proceeds, and to what extent such a shift moves the country toward a low-carbon society by 2100. Paradoxically, diversity measured by the HI declines when the dependence on fossil fuels declines, because the three types of fossil fuels contribute to the “diversity” of energy sources and the use of a specific non-fossil energy source increases. However, such declines in diversity are derived from a decrease in the use of fossil fuels, most of which are imported in China. Thus, overall, it is demonstrated that climate mitigation alters energy structure and contributes to improved energy security, although the diversity of energy sources appears to suggest otherwise.

In this study, global-scale emission trading is taken into account, meaning that world GHG emissions are reduced in a cost-efficient manner. If it is not assumed, and emissions reduction are implemented in each country or emissions trading is introduced at the regional level, such as emissions trading in the European Union, GHG emissions in China will increase, since China acts as a seller of emissions permits in the global-scale emissions trading system. This is due to its lower marginal abatement cost as compared to the global average. Thus, the demand for fossil fuels will increase, while the share of renewables and nuclear energy will decrease in this situation. From this perspective, it is expected that fossil fuel imports will increase during this century, while the influence on the diversity of energy sources can be different by scenario (more diversified in the case that the share of fossil fuels is relatively small and less diversified in the case that the share is relatively large). Consequently, energy security can be somewhat worse when not introducing global-scale emissions trading.

## Concluding Remarks

In this study, the impact of climate mitigation policies on energy structure and energy security was analyzed by using a CGE model. In the analysis, the author used RCP-based scenarios for the policy scenarios and compared them with a reference scenario.

To reduce GHG emissions, China needs to shift its energy structure from fossil fuel dominance to nuclear power and renewables, including biomass power. Among fossil fuels, coal must be significantly reduced, followed by oil and natural gas. The lower the target of allowable emissions, the larger the shifts from fossil fuels must be. The study also reveals that such shifts will improve energy self-sufficiency and are consequently effective from the viewpoint of energy security. However, the impact on diversity of energy sources under climate mitigation scenarios differs by scenario.

China was a net exporter of coal in the base year, but it soon became a net importer. The diversity of energy sources, as measured by the HI, worsened in the reference scenario because of a large increase in fossil fuel use, particularly coal. Introducing the policy scenarios to reduce GHG emissions resulted in significant improvement in the diversity index in response to an increasing share of renewables and a decreasing share of coal. A decrease in fossil fuel imports was also observed.

The main purpose of climate mitigation is obviously to reduce GHG emissions and avoid further climate change. This study indicates that energy security improvement is achieved simultaneously under climate mitigation policies. Although introducing climate mitigation policies has a negative effect on economic growth in general [56], energy security improvements will contribute to the economy by reducing procurement risks.

Reduction in diversity of energy sources is a potential consequence of reducing net imports of fossil fuels and increased reliance on domestic and semi-domestic energy sources. Dependence on only a few energy sources poses risks. For example, nuclear power plants are susceptible to severe accidents, as demonstrated in Japan in 2011. Renewables such as solar and wind power are variable because they are affected by meteorological and climatic factors [22–24]. Climate predictions and weather forecasts for planning of the resources [27], reserve capacity and energy storage solutions [61], and a diversity of power sources are crucial components of efforts to achieve a low-carbon society.

In this study, diversification of the origin of fossil fuel imports was not analyzed, because the CGE model does not disaggregate the regions (Table 1). For example, the countries of the oil-producing Middle East are not disaggregated. Furthermore, the country risks of the origin of fossil fuel imports were not included in evaluating energy security, which was investigated in a few previous studies [33–34], due to the model limitation. These important aspects of energy security in China remain to be investigated.

## Supporting Information

S1 Dataset Data used in Figs 2–7. (XLSX)
